# RGMa promotes dedifferentiation of vascular smooth muscle cells into a macrophage-like phenotype in vivo and in vitro

**DOI:** 10.1016/j.jlr.2022.100276

**Published:** 2022-09-09

**Authors:** Xiaofan Yuan, Hongmei Xiao, Qingzhe Hu, Guanru Shen, Xinyue Qin

**Affiliations:** Department of Neurology, The First Affiliated Hospital of Chongqing Medical University, Chongqing, China

**Keywords:** RGMa, Slug, atherosclerosis, carotid artery ligation, neointima, dedifferentiation, VSMCs, oxidized LDL, ApoE, apolipoprotein E, BMP, bone morphogenetic protein, CCA, common carotid artery, EMT, epithelial-to-mesenchymal transition, EndMT, endothelial-to-mesenchymal transition, HFD, high-fat diet, NC, negative control, ox-LDL, oxidized LDL, RGMa, repulsive guidance molecule a, SMAD, Small Mothers Against Decapentaplegic, TGF-β, transforming growth factor beta, VSMCs, vascular smooth muscle cells

## Abstract

Repulsive guidance molecule a (RGMa) is a glycosylphosphatidylinositol-anchored glycoprotein that has been demonstrated to influence inflammatory-related diseases in addition to regulating neuronal differentiation and survival during brain development. However, any function or mechanism of RGMa in dedifferentiation of contractile vascular smooth muscle cells (VSMCs) during inflammatory-related atherosclerosis is poorly understood. In the current study, we found that RGMa is expressed in VSMCs-derived macrophage-like cells from the fibrous cap of type V atherosclerotic plaques and the neointima of ligated carotid artery in ApoE^−/−^ mice. We determined levels of RGMa mRNA and protein increased in oxidized LDL (ox-LDL)-induced VSMCs. Knockdown of RGMa, both in vivo and in vitro, inhibited the dedifferentiation of ox-LDL-induced VSMCs and their ability to proliferate and migrate, reduced the thickness of the neointima after ligation of the left common carotid artery in ApoE^−/−^ mice. Additionally, we show RGMa promoted the dedifferentiation of VSMCs via enhancement of the role of transcription factor Slug. Slug knockdown reversed the dedifferentiation of ox-LDL-induced VSMCs promoted by RGMa overexpression. Thus, inhibition of RGMa may constitute a therapeutic strategy for atherosclerotic plaques prone to rupture and restenosis following mechanical injury.

Ischemic attacks often result from artery-to-artery emboli following the rupture of atherosclerotic plaque rather than from hypoperfusion due to luminal stenosis (1). The ruptured plaques are characterized by large necrotic lipid-rich cores covered by a thin fibrous cap, increased macrophages infiltration, intraplaque hemorrhage formation, and thrombosis (2). Vascular smooth muscle cells (VSMCs) are the major component of plaque cells and, together with the extracellular matrix, contribute to various processes throughout all stages of atherosclerosis (3, 4, 5). VSMCs involved in carotid artery injury and atherosclerosis models have been subjected to lineage tracing studies to determine their origins and fates. Such studies have demonstrated that VSMCs are not terminally differentiated and can undergo marked phenotypic changes, forming foam cells, macrophage-like cells, osteoblast-like cells, and mesenchymal stem-like cells during pathogenesis (6, 7, 8). The function of VSMCs varies dramatically, depending on the nature of phenotypic transitions (9). It is noteworthy that VSMCs-derived macrophages feature in all stages of atherosclerosis, from initiation of plaques and lesion progression, to necrosis leading to rupture of susceptible plaques (10). Therefore, the process by which VSMCs dedifferentiate into macrophage-like cells presents a therapeutic target to control the stability of life-threatening plaques in atherosclerosis and for treatment of the neointima in injury-induced diseases.

Repulsive guidance molecule a (RGMa) is a glycosylphosphatidylinositol-anchored membrane glycoprotein that was initially observed to guide retinal axons during chick embryogenesis and to regulate neuronal differentiation during brain development (11, 12, 13). There is growing acknowledgement of RGMa in the occurrence and development of inflammatory-related diseases (14). RGMa is also a high-affinity co-receptor of bone morphogenetic proteins (BMPs) and has been demonstrated to influence iron homeostasis and endochondral bone development by activating the Small Mothers Against Decapentaplegic (SMAD) signaling pathway (15). It is noteworthy that circulating BMP9 and BMP10 have hormone-like activity and may bind to activin receptor-like kinase receptors to stimulate the formation of contractile VSMCs (16). In previous work from our laboratory, RGMa was found to be expressed in VSMCs and astrocytes and to form a complex with activin receptor-like kinase 5 and SMAD2/3 to facilitate phosphorylation in rat middle cerebral artery occlusion/reperfusion models (17). Functional polymorphisms of the RGMa promoter were also correlated with the cerebrovascular atherosclerosis burden in patients diagnosed with acute ischemic cerebrovascular events (18). However, any role of RGMa in VSMCs during atherosclerosis and structural remodeling after arterial injury is unclear.

The zinc-finger transcription factor, Snail 2/Slug, has a pivotal role in epithelial-to-mesenchymal transition (EMT) and endothelial-to-mesenchymal transition (EndMT) (19, 20, 21, 22). The process of EMT is known to contribute to increased metastasis during malignant diseases (19, 20), and EndMT promotes structural remodeling during vascular diseases (21, 22). Transforming growth factor beta (TGF-β) activates Slug to form a transcriptional complex with SMAD2/3 and promotes EMT/EndMT-related diseases (19, 20, 21, 22, 23, 24). The processes of EMT/EndMT share some similarities with the phenotypic dedifferentiation of VSMCs in atherosclerosis and injury-induced neointimal diseases, in which the expression of differentiated VSMCs markers decreases and markers related to dedifferentiated phenotypes increase. Slug has been found to be involved in VSMCs transdifferentiation induced by platelet-derived growth factor-BB during atherosclerosis (25). Subsequent to our previous studies, we propose that RGMa, as a co-receptor of TGF-β superfamily, participates in the regulation of Slug to promote the dedifferentiation of VSMCs.

Therefore, the current study investigated the role of RGMa in atherosclerosis and injury-induced remodeling and explored potential mechanisms of VSMCs dedifferentiation in vivo and in vitro.

## Materials and methods

Mice lacking apolipoprotein E (ApoE^−/−^) and Sprague-Dawley rats were supplied by the Laboratory Animal Center of Chongqing Medical University (Chongqing, China). All animal studies were approved by the Ethics Committee of the First Affiliated Hospital of Chongqing Medical University.

### Animal models

#### Atherosclerosis model

Male ApoE^−/−^ mice (8 weeks old) were fed a high-fat diet (HFD; catalog no.: D12108C; Research Diets) (26) or a standard diet for 16 weeks before they were anesthetized and euthanized. Aortas, including thoracic and abdominal segments, were dissected, fixed with 4% paraformaldehyde for Oil Red O (catalog no.: G1261; Solarbio, China) staining, according to the manufacturer's instructions. Aortic arches were embedded in paraffin and cut into 5 μm-thick serial sections for H&E staining, as described previously (27). The aortas thickness of intimal and medial areas were measured using ImageJ software (National Institutes of Health).

#### Common carotid artery ligation

ApoE^−/−^ mice were subjected to complete common carotid artery (CCA) ligation on the left side, as described previously (27, 28). Briefly, the left CCA proximal to the carotid bifurcation was dissected and ligated using 6-0 silk. In some animals, 30 μl of 30% F127 Pluronic gel (catalog no.: P2245; Sigma, Germany) containing 5 μl RGMa-specific recombinant adenovirus (Ad-shRGMa, 2 × 10^10^ plaque-forming unit/ml; Tsingke Biotechnology Co, Ltd, China) or GFP carrier recombinant adenovirus (Ad-NC, 2 × 10^10^ plaque-forming unit/ml) were applied to the carotid artery by periadventitial administration immediately after ligation. The sequence of shRGMa was CAACTACACTCACTGCGGCCT. In the sham-operated group, the CCA was exposed but not ligated. CCAs were harvested at 21 days after ligation with removal of a 5 mm long artery proximal to the ligation site. Dissected arteries were cut into two segments and embedded in either paraffin or OCT (Tissue-Tek).

### Culture of primary VSMCs and transfection

#### Cell culture

Primary VSMCs were isolated from the aortas of Sprague-Dawley rats (males, weight: 100–150 g), as described in the literature (29). Cells were cultured in DMEM (catalog no.: 88287; Gibco, MA) supplemented with 10% FBS (catalog no.: C0235; Gibco, Australia) and 1% penicillin/streptomycin (catalog no.: C0222; Beyotime, China). Cells were cultured at 37°C in a humidified atmosphere with 5% CO_2_ and used between passages 3 and 6.

#### Cell transfection

The RGMa sequence was inserted into a pcDNA3.1(+) vector (GenePharma, China) to generate overexpression (pcDNA-RGMa) and into the empty pcDNA3.1(+) vector as a negative control (pcDNA-NC). Transfection into rat VSMCs was performed via advanced Transfection Reagent (catalog no.: AD600150; ZetaLife), according to the manufacturer’s protocol. siRNAs against rat RGMa (si-RGMa), Slug (si-Slug), and NC (si-NC) were designed and synthesized by GenePharma (Shanghai, China). *In vitro* transfection with siRNAs were performed using Lipofectamine RNAiMax Reagent (catalog no.: 13778030; Invitrogen). Si-RNA sequences are as follows: si-RGMa: GUAGACGUUUGGCCAUGUATT (5′ to 3′) and UACAUGGCCAAACGUCUACTT (3′ to 5′); si-Slug: GCUGAGAAGUUU-CAGUGCAAUUUAUTT (5′ to 3′) and AUAAAUUGCACUGAAACUUCUCAGC TT (3′ to 5′).

### Cell migration assay

#### Wound healing

VSMCs were grown in 6-well plates until 70–80% confluence was reached, at which point cells were treated with si-NC or si-RGMa overnight, followed by serum starvation for 24 h. Confluent cells were scratched using a 200 μl pipette tip. Cells were cultured in the presence or the absence of 10 μg/ml oxidized LDL (ox-LDL; catalog no.: IO300; Solarbio, China) for 24 h, and cell migration into the scratched areas was monitored at 0 and 24 h after assay by microscopy (Leica, Germany).

#### Transwell assay

Transwell migration assays were performed using an 8 μm chamber (catalog no.: 3422; Corning). VSMCs were transfected with si-RGMa or si-NC as described above, detached using 0.25% trypsin (catalog no.: C0201; Beyotime) and seeded into the upper chamber. The lower chamber contained 500 μl medium with or without 10 μg/ml ox-LDL to induce VSMCs migration. After 12 h, cells in the upper chamber were wiped with a cotton swab, and migrated cells on the underside were fixed and stained with 0.1% crystal violet (catalog no.: C0121; Beyotime). Image-Pro Plus 6.0 (National Institutes of Health) software was used to measure the migration areas and to count the number of VSMCs across the Transwell chambers.

### Cell proliferation assay

VSMCs proliferation was measured by Cell Counting Kit-8 (catalog no.: C0037; Beyotime), according to the manufacturer’s instructions. After transfection with si-RGMa or si-NC, VSMCs were seeded into a 96-well plate and starved in serum-free medium overnight. Cells were incubated with or without 10 μg/ml ox-LDL for 24 h, after which 10 μl solution was added to each well, followed by incubation at 37°C for 2 h. Absorbances at 450 nm were measured using a microplate reader (Heales, China).

### Immunofluorescence

Primary rat VSMCs and arterial sections were collected for immunofluorescence staining. Briefly, VSMCs were fixed in 4% paraformaldehyde for 30 min, and arterial sections were repaired with antigen retrieval sodium buffer. Cells or arterial sections were permeabilized with 0.4% Triton X-100 (catalog no.: ST795; Beyotime) and blocked with 10% donkey serum (catalog no.: SL038; Solarbio, China) at room temperature. Samples were incubated with primary antibodies against RGMa (1:50 dilution; catalog no.: sc-393046; Santa Cruz), CD68 (1:100 dilution; catalog no.: ab201340; abcam, UK), SM22α (1:200 dilution; catalog no.: ab14106; abcam), α-SMA (1:200 dilution; catalog no.: 19245; CST, UK), and Slug (1:50 dilution; catalog no.: sc-166476; Santa Cruz) at 4°C overnight, followed by secondary antibodies Alexa Fluor 488 (1:200 dilution; catalog no.: SA00013-5; Proteintech, China) and 555 (1:100 dilution; catalog no.: A0460; Beyotime) for 1 h at room temperature. Nuclei were counterstained with 4′,6-diamidino-2-phenylindole (catalog no.: C1002; Beyotime) for 10 min and protected by antifade mounting medium. Triple immunofluorescence staining was performed according to the manufacturer's instructions (catalog no.: AFIHC024; China). Images were captured using a confocal laser scanning microscope (Andor, UK) and analyzed by ImageJ software.

### Quantitative real-time PCR

Total RNA was isolated from VSMCs using Trizol reagent (catalog no.: 9108; Takara), and 1 μg aliquots reverse-transcribed into complementary DNA (catalog no.: PR047; Takara). Quantitative real-time PCR using SYBR Green reagent (catalog no.: PR802A; Takara) was performed according to the manufacturer’s protocols. Relative expression of mRNA was normalized to β-actin level.

### Western blot analysis

Western blot analysis was performed as previously described (30). Total proteins were extracted from primary VSMCs or arterial tissues by RIPA lysis buffer (catalog no.: PM0013B; Beyotime) and quantified using bicinchoninic acid protein assay kit (catalog no.: P0009; Beyotime). Equal amounts of total protein were loaded onto SDS-PAGE gels and, following separation, were transferred to 0.45 μm PVDF membranes (Millipore, Billerica, MA). Membranes were blocked with 5% nonfat milk for 1 h and incubated with primary antibodies against RGMa (1:10,000 dilution; catalog no.: ab169761; abcam), CD68 (1:100 dilution; catalog no.: ab201340; abcam), SM22α (1:2,000 dilution; catalog no.: ab14106; abcam), α-SMA (1:1,000 dilution; catalog no.: 19245; CST), Slug (1:500 dilution; catalog no.: ab180714; abcam), neogenin (1:1,000 dilution; catalog no.: NBP1-89651; Novus), p-ERK1/2 (1:1,000 dilution; catalog no.: 9102; CST), ERK1/2 (1:1,000 dilution; catalog no.: 9101; CST), p-MEK1/2 (1:1,000 dilution; catalog no.: 9154; CST), or MEK1/2 (1:1,000 dilution; catalog no.: 9122; CST) at 4°C overnight. Goat anti-mouse (1:5,000 dilution; catalog no.: SA00001-1; Proteintech, China) or goat anti-rabbit (1:5,000 dilution; catalog no.: SA00001-2; Proteintech, China) secondary antibodies were added for incubation for 1 h at room temperature. Images were captured and quantified by Fusion FX5 image analysis system (Vilber Lourmat, France).

### Statistical analysis

Statistical analysis was performed in GraphPad Prism 9.0 (GraphPad Software, Inc) and SPSS 26.0 (IBM) software. All data represent results from at least three independent experiments. Student’s *t*-test and one-way repeated-measures ANOVA were used for comparisons between groups and subgroups. Chi-square and Mann-Whitney U tests were used for nonparametric data. A difference was considered statistically significant when *P* < 0.05.

## Results

### RGMa is expressed in the dedifferentiated VSMCs from the atherosclerotic plaque

**Fig. 1:**
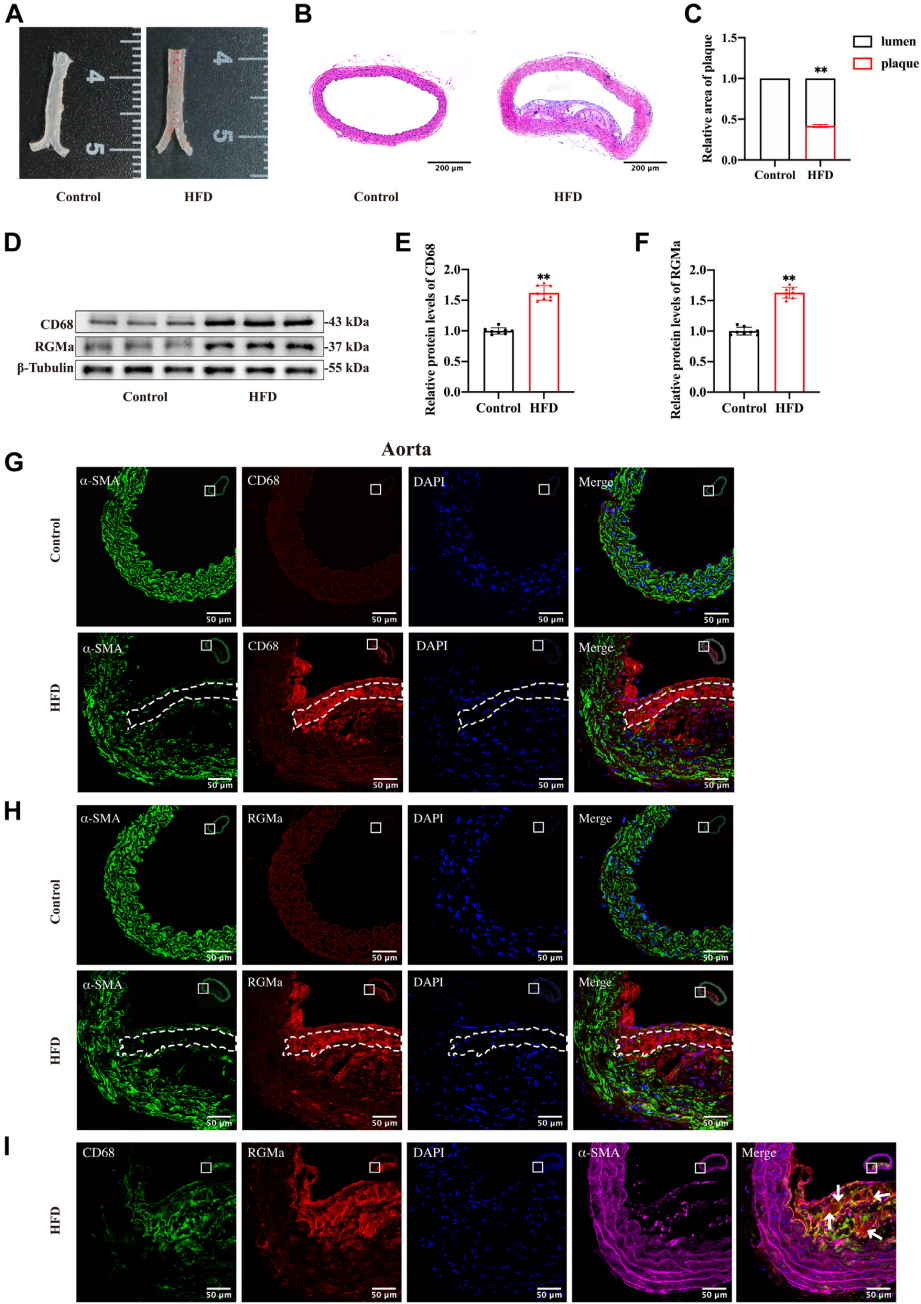
RGMa is expressed in the macrophage-like VSMCs of type V atherosclerotic plaques. A: Representative photomicrographs of aortic Oil Red O staining of ApoE^−/−^ mice after feeding 16 weeks on the standard diet or HFD; n = 4. B, C: Type V atherosclerotic lesion deposited in the aortic wall of HFD-fed ApoE^−/−^ mice. Τhe standard diet-fed mice had no significant atherosclerotic plaques formation in the aortas. n = 5; ∗∗*P* < 0.01; the scale bar represents 200 μm. D–F: Western blot analysis (D) and quantification for the relative protein levels of RGMa (E) and CD68 (F) in the aortas of HFD or standard diet-fed ApoE^−/−^ mice. n = 8; ∗∗*P* < 0.01. G, H: Immunofluorescence staining of CD68 (red), α-SMA (green), and RGMa (red) in the aortic cross-sections of HFD and standard diet-fed ApoE^−/−^ mice. Nuclei were stained with DAPI (blue). The region of interest represents the media layer of atherosclerotic fibrous cap. n = 5; the scale bar represents 50 μm. I: Triple immunofluorescence staining of CD68 (green), RGMa (red), and α-SMA (magenta) in the aortic cross-sections of HFD-fed ApoE^−/−^ mice. Nuclei were stained with DAPI (blue). RGMa was expressed in the CD68-positive VSMCs resident in the media layer of atherosclerotic fibrous cap (white arrows). n = 5; the scale bar represents 50 μm. DAPI, 4′,6-diamidino-2-phenylindole.

ApoE^−/−^ mice fed an HFD for 16 weeks had atherosclerotic plaques formation, visualized by Oil Red O staining of the arterial wall. By contrast, control ApoE^−/−^ mice that were fed a standard diet showed no significant lipid deposition in the aortas (Fig. 1A). H&E staining of aortic cross-sections from HFD-fed mice indicated the formation of type V atherosclerotic lesion, characterized by fibroatheromas containing necrotic lipid-rich cores with the development of prominent fibrous cap (Fig. 1B, C). Western blot analysis showed that RGMa and CD68 proteins were both upregulated in the aortas of HFD-fed ApoE^−/−^ mice compared with controls (Fig. 1D–F). The middle layer of the fibrous cap was characterized by decreased numbers of α-SMA-positive VSMCs and increased numbers of CD68-positive VSMCs detected by immunofluorescence (Fig. 1G). The frequent phenotypic dedifferentiation of VSMCs in this region has been reported previously (17). RGMa was detected in the CD68-positive but not in the α-SMA-positive VSMCs resident in the media of atherosclerotic fibrous cap (Fig. 1H, I). These results demonstrate the possibility that RGMa may be involved in the formation of atherosclerotic plaques induced by feeding an HFD in ApoE^−/−^ mice. The process may involve the dedifferentiation of contractile VSMCs resident in the middle layer of fibrous cap into a macrophage-like phenotype, thereby increasing the vulnerability of atherosclerotic plaques.

### RGMa upregulation correlates with the dedifferentiation of contractile VSMCs in vitro and in vivo

**Fig. 2:**
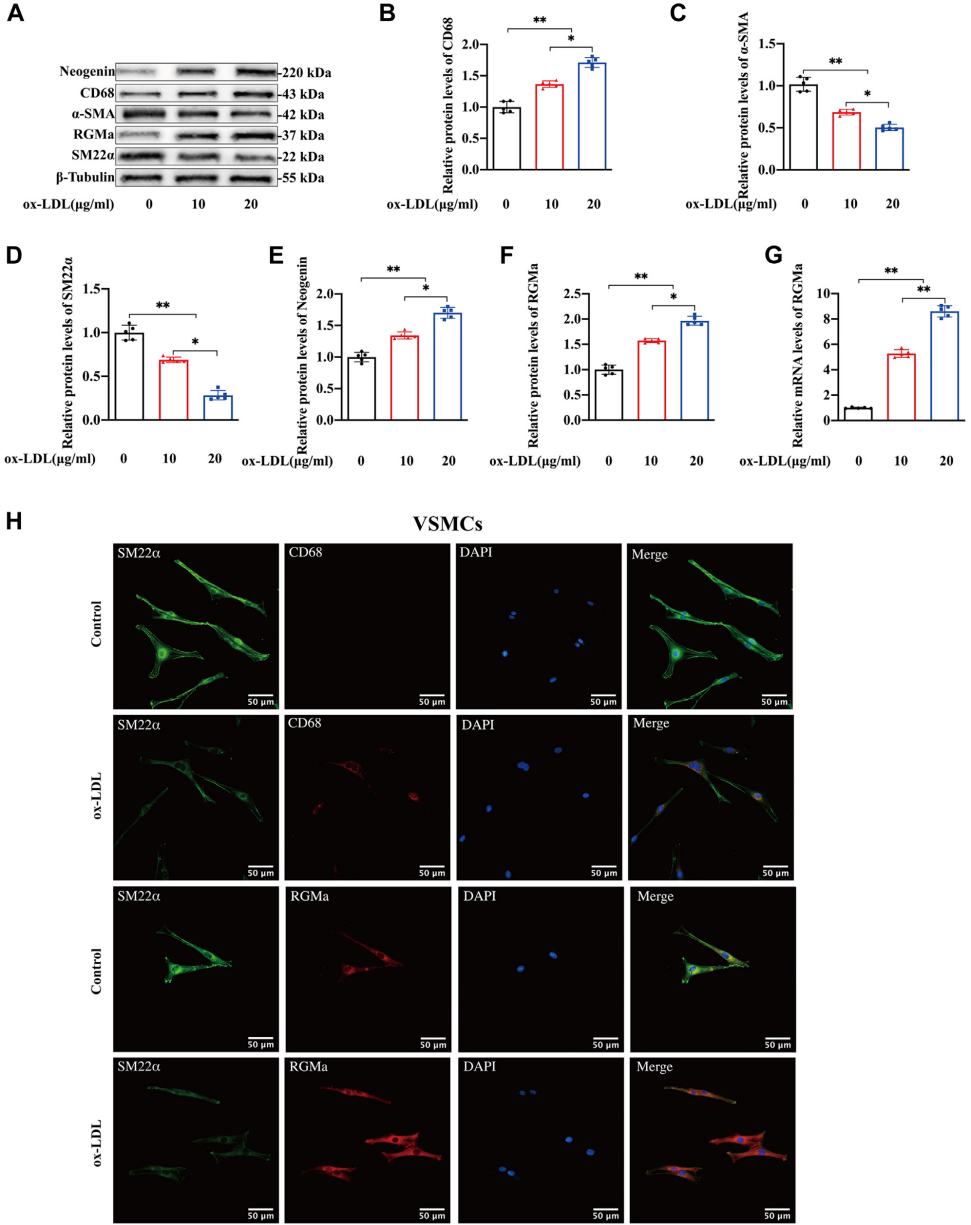

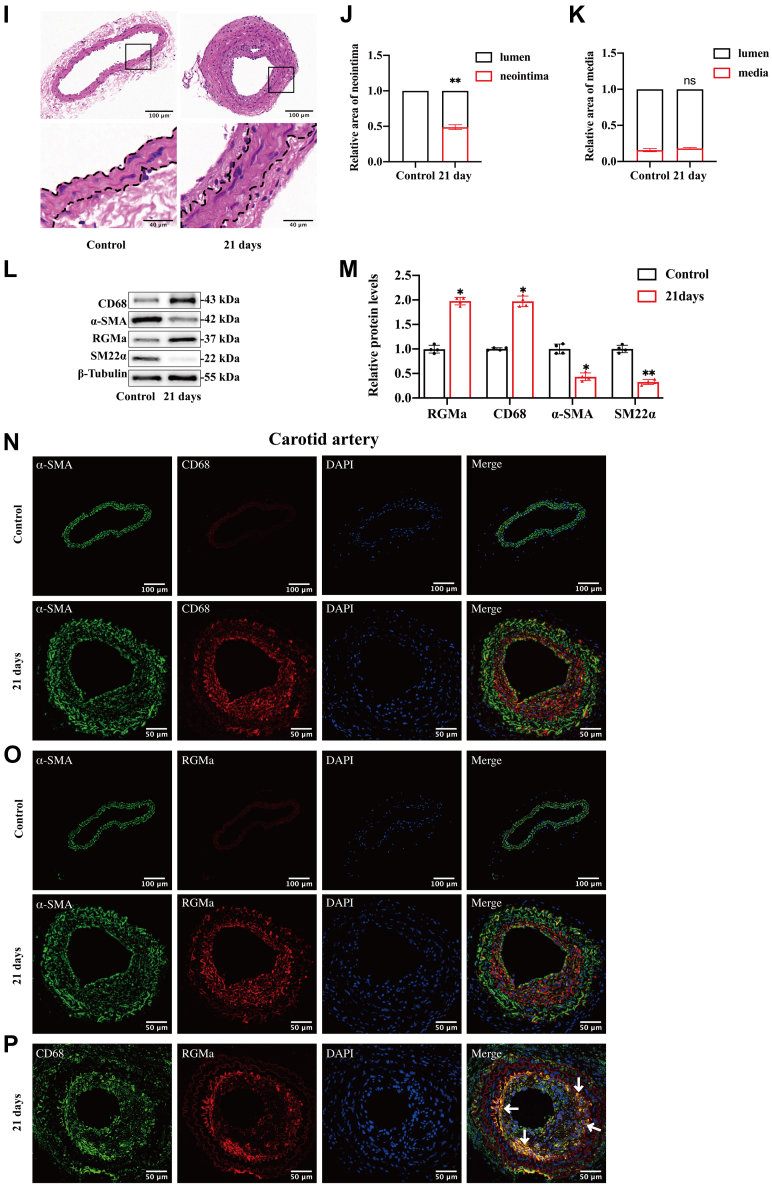
The expression of RGMa upregulates in the macrophage-like VSMCs and the neointima after carotid artery ligation. A–G: Western blot analysis (A) and quantification for the relative protein expression of CD68 (B), α-SMA (C), SM22α (D), neogenin (E), RGMa (F), and the relative mRNA levels of RGMa (G) in the primary rat VSMCs cultured with gradient ox-LDL (0, 10, and 20 μg/ml) for 24 h. n = 5; ∗*P* < 0.05, ∗∗*P* < 0.01. H: Immunofluorescence staining of CD68 (red), SM22α (green), and RGMa (red) in the primary rat VSMCs cultured with or without ox-LDL (10 μg/ml) for 24 h. Nuclei were stained with DAPI (blue). n = 3; the scale bar represents 50 μm. I–K: Representative photomicrographs of H&E staining in the sham and ligated carotid arteries for 21 days of ApoE^−/−^ mice (I). Quantification for the relative area of neointima (J) and media (K) between the above groups. n = 5; ∗∗*P* < 0.01, ns indicates not significant; the scale bar represents 100 μm and 40 μm. L, M: Western blot analysis (L) and quantification (M) for the relative protein expression of RGMa, CD68, α-SMA, and SM22α in the sham and ligated carotid arteries for 21 days of ApoE^−/−^ mice. n = 4; ∗*P* < 0.05; ∗∗*P* < 0.01. N, O: Immunofluorescence staining of CD68 (red), α-SMA (green), and RGMa (red) in the sham and ligated carotid arteries for 21 days of ApoE^−/−^ mice. Nuclei were stained with DAPI (blue). n = 5; the scale bar represents 50 and 100 μm. P: Immunofluorescence staining of CD68 (green) and RGMa (red) in the ligated carotid arteries for 21 days of ApoE^−/−^ mice. Nuclei were stained with DAPI (blue). RGMa was observed in the CD68-positive VSMCs of neointima in the ligated carotid arteries (white arrows); n = 5; the scale bar represents 50 μm. DAPI, 4′,6-diamidino-2-phenylindole.

As part of an investigation into RGMa and its receptor, expression of RGMa and neogenin in cultured rat primary VSMCs was measured. After 24 h of treatment with 0, 10, or 20 μg/ml ox-LDL in DMEM, cells gradually dedifferentiated into macrophage-like VSMCs, manifested by a decrease in expression of the contractile markers, SM22α and α-SMA, and an increase in the macrophage marker, CD68 (Fig. 2A–D). These changes were accompanied by increased levels of RGMa and neogenin (Fig. 2E–G). Similar trends in RGMa, SM22α, and CD68 levels were confirmed by immunofluorescence staining assays (Fig. 2H).

A mouse model designed to demonstrate the dedifferentiation of contractile VSMCs was constructed by complete ligation of the CCA for 21 days in ApoE^−/−^ mice. H&E staining of carotid artery cross-sections showed an increased area of neointima when compared with the sham-operated carotid arteries (Fig. 2I, J). And the relative area of media showed no change between the sham and ligated carotid arteries (Fig. 2K). Expression of RGMa and CD68 proteins were increased in the CCA after ligation, whereas SM22α and α-SMA proteins were decreased (Fig. 2L, M). Immunofluorescence staining showed that increased RGMa and CD68, and decreased α-SMA were observed in the neointima but not in the media of ligated carotid arteries (Fig. 2N, O). And the increased RGMa co-localized with CD68-positive VSMCs resident in the neointima of ligated arteries (Fig. 2P). In summary, the foregoing results indicate that the upregulated RGMa is associated with the dedifferentiation of contractile VSMCs both in vivo and in vitro.

### RGMa knockdown inhibits the dedifferentiation of contractile VSMCs and neointima formation

**Fig. 3:**
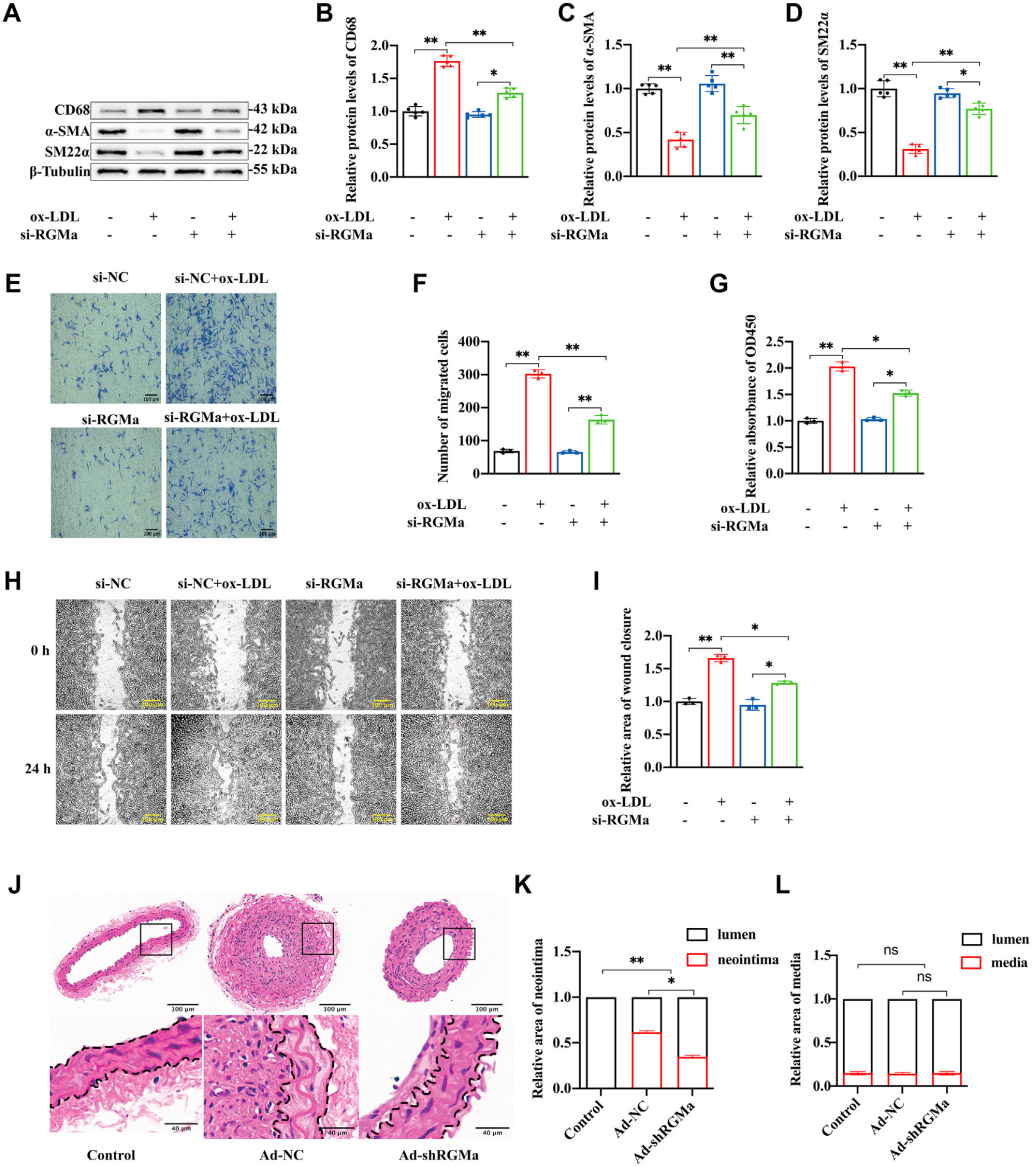
RGMa mediates the dedifferentiation, proliferation, and migration of VSMCs and the neointima formation after arterial injury. A–D: Western blot analysis (A) and quantification for the relative expression of CD68 (B), α-SMA (C), and SM22α (D) in the primary rat VSMCs transfected with or without si-RGMa, followed by or not ox-LDL (10 μg/ml for 24 h) treatment. n = 5; ∗*P* < 0.05, ∗∗*P* < 0.01. E, F: The migration ability of VSMCs determined by a transwell chamber assay. The VSMCs were cultured in different conditions (si-NC, si-NC + ox-LDL, si-RGMa, and si-RGMa + ox-LDL). n = 3; ∗∗*P* < 0.01; the scale bar represents 100 μm. G: Same treated VSMCs (si-NC, si-NC + ox-LDL, si-RGMa, and si-RGMa + ox-LDL) were subjected to Cell Counting Kit-8 assay to detect the proliferation ability of VSMCs. n = 3; ∗*P* < 0.05; ∗∗*P* < 0.01. H, I: Wound healing assay was performed on ox-LDL-induced VSMCs treated with si-RGMa or si-NC. n = 3; ∗*P* < 0.05; ∗∗*P* < 0.01; the scale bar represents 100 μm. J: Representative photomicrographs of H&E staining in the control, Ad-NC-, and Ad-shRGMa-treated carotid arteries 21 days after ligation in ApoE^−/−^ mice. n = 6; the scale bar represents 100 and 40 μm. K, L: Quantification for the relative area of the neointima (K) and media (L) among the control, Ad-NC-, and Ad-shRGMa-treated carotid arteries 21 days after ligation in ApoE^−/−^ mice. n = 5; ∗*P* < 0.05; ∗∗*P* < 0.01, ns indicates not significant.

**Supplemental Fig. S1:**
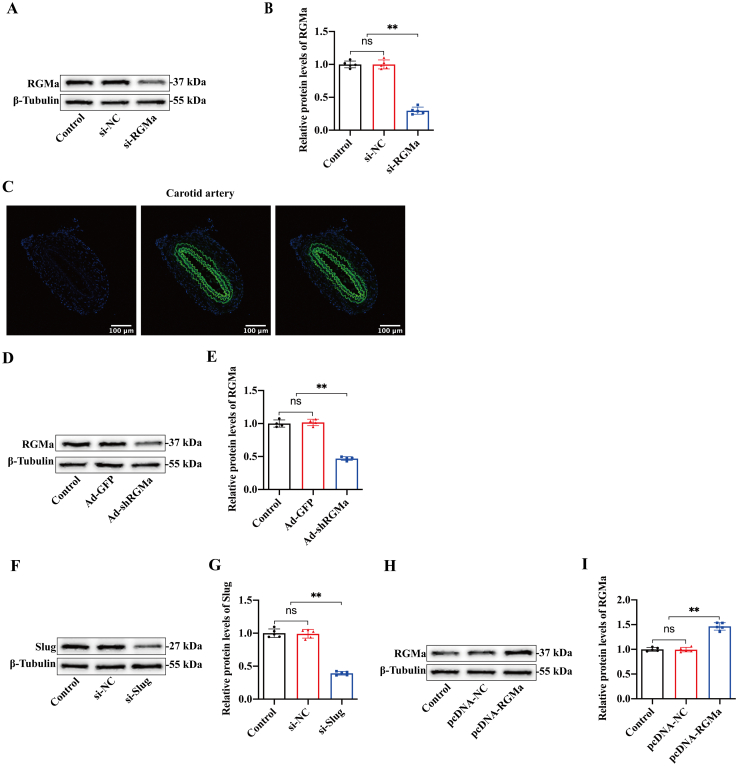
A, B: Western blot analysis (A) and quantification for the relative expression of RGMa (B) in the primary rat VSMCs transfected with si-RGMa or si-NC. n = 5; ∗∗*P* < 0.01, ns indicates not significant. C: The left carotid artery of ApoE^−^/^−^ mice treated with Ad-shRGMa for 14 days, and pictured by immunofluorescence. Nuclei were stained with DAPI (blue). n = 4; Scale bar: 100 μm. D, E: Western blot analysis (D) and quantification for the relative expression of RGMa (E) in the Ad-shRGMa or Ad-NC treated carotid arteries of ApoE^−^/^−^ mice. n = 4; ∗∗*P* < 0.01, ns indicates not significant. F, G: Western blot analysis (F) and quantification for the relative expression of Slug (G) in the primary rat VSMCs transfected with si-Slug or si-NC. n = 5; ∗∗*P* < 0.01, ns indicates not significant. H, I: Western blot analysis (H) and quantification for the expression of RGMa (I) in the primary rat VSMCs transfected with pcDNA-RGMa or pcDNA-NC. n = 5; ∗∗*P* < 0.01, ns indicates not significant.

Expression of RGMa in rat primary VSMCs was silenced by si-RGMa, and the expected reduction of RGMa detected by Western blot analysis (supplemental Fig. S1A, B). RGMa knockdown reversed the ox-LDL-stimulated events in VSMCs. Western blot analysis showed increased levels of the VSMCs contractile markers, SM22α and α-SMA, and decreased levels of the macrophage-like marker, CD68, in ox-LDL-stimulated VSMCs in which RGMa was silenced (Fig. 3A-D). Also, Wound healing, Transwell, and Cell Counting Kit-8 assays showed that the ability of proliferation and migration was reduced in the ox-LDL-induced VSMCs in which RGMa was silenced (Fig. 3E-I). Thus, RGMa knockdown inhibited proliferation, migration, and dedifferentiation of rat VSMCs stimulated by ox-LDL.

The left CCA of ApoE^−/−^ mice was ligated, followed by adventitial administration of Ad-shRGMa or Ad-NC mixed in Pluronic gel for 14 days. The Ad-shRGMa resulted in knockdown of the RGMa expression in carotid arteries as shown by immunofluorescence and Western blot analysis (supplemental Fig. S1C–E). Morphometric analysis of carotid arteries stained by H&E in the sham, Ad-NC, and Ad-shRGMa groups was performed on the 21st day post-ligation (Fig. 3J). And the results showed that the relative area of neointima was lower and the area of the lumen was higher in the ligated arteries from Ad-shRGMa-treated carotid arteries when compared with Ad-NC-treated carotid arteries (Fig. 3K). However, no difference in the area of media was found among sham-, Ad-NC-, and Ad-shRGMa-treated carotid arteries (Fig. 3L). Thus, knockdown of RGMa inhibited neointima formation after CCA ligation.

### Slug is involved in the ox-LDL-induced dedifferentiation of VSMCs

**Fig. 4:**
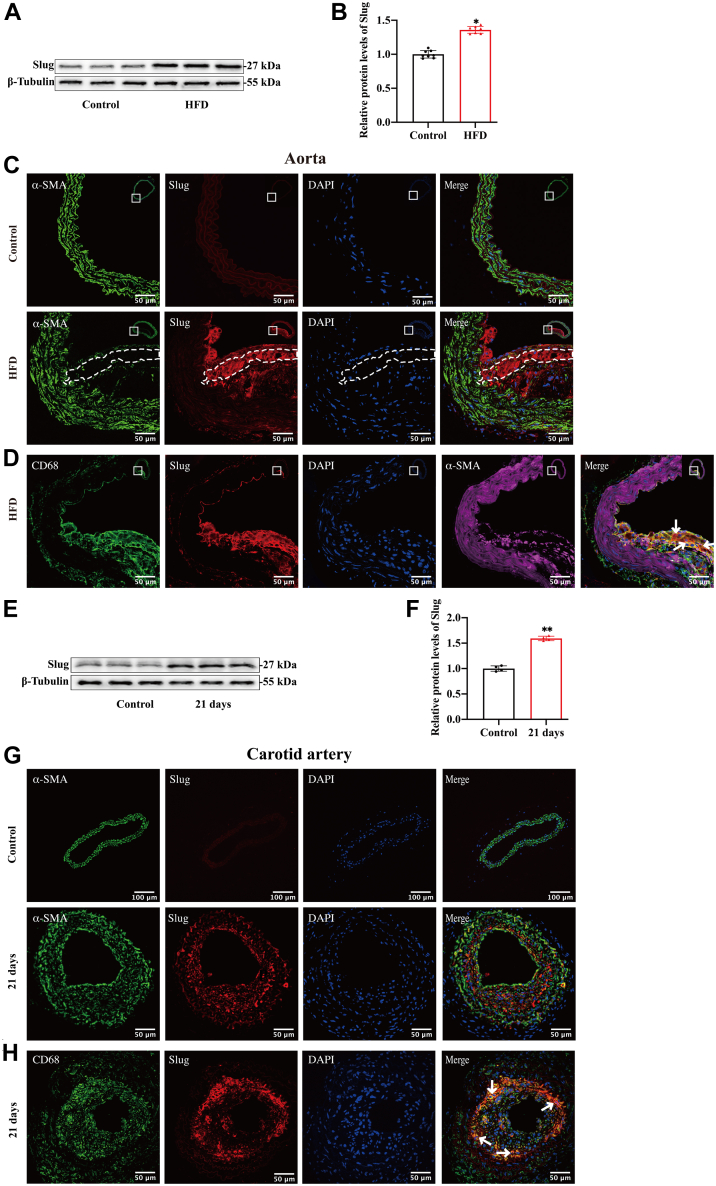

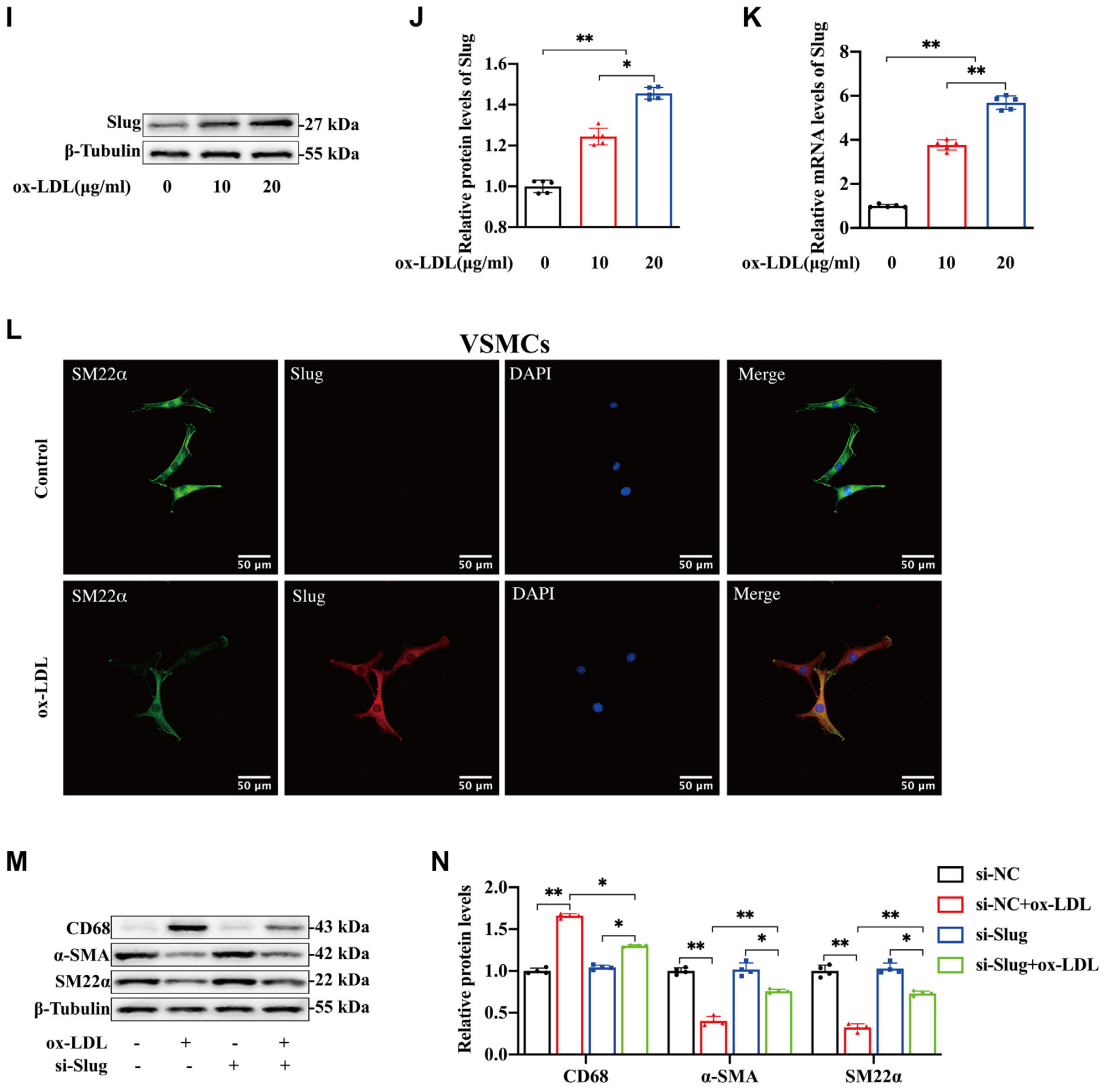
Slug is involved in the dedifferentiation of VSMCs. A, B: Western blot analysis (A) and quantification for relative expression of Slug (B) in the aortas of HFD or standard diet-fed ApoE^−/−^ mice. n = 8; ∗*P* < 0.05. C: Immunofluorescence staining of Slug (red) and α-SMA (green) in the aortic cross-sections of HFD and standard diet-fed ApoE^−/−^ mice. Nuclei were stained with DAPI (blue). The region of interest represents the media layer of atherosclerotic fibrous cap. n = 5; the scale bar represents 50 μm. D: Triple immunofluorescence staining of CD68 (green), Slug (red), and α-SMA (magenta) in the aortic cross-sections of HFD ApoE^−/−^ mice. Nuclei were stained with DAPI (blue). Slug was detected in the CD68-positive VSMCs resident in the media layer of atherosclerotic fibrous cap (white arrows). n = 5; the scale bar represents 50 μm. E, F: Western blot analysis (E) and quantification for the relative protein expression of Slug (F) in the sham and ligated carotid arteries of ApoE^−/−^ mice. n = 4; ∗∗*P* < 0.01. G: Immunofluorescence staining of α-SMA (green) and Slug (red) in the sham and ligated carotid arteries for 21 days of ApoE^−/−^ mice. Nuclei were stained with DAPI (blue). n = 5; the scale bar represents 50 and 100 μm. H: Immunofluorescence staining of CD68 (green) and Slug (red) in the ligated carotid arteries for 21 days of ApoE^−/−^ mice. Nuclei were stained with DAPI (blue). Slug was observed in the CD68-positive VSMCs of neointima in the ligated carotid arteries (white arrows); n = 5; the scale bar represents 50 μm. I–K: Western blot analysis (I) and quantification for the relative protein expression of Slug (J) and mRNA expression of Slug (K) in the primary rat VSMCs cultured with gradient ox-LDL (0, 10, and 20 μg/ml) for 24 h. n = 5; ∗*P* < 0.05, ∗∗*P* < 0.01. L: Immunofluorescence staining of Slug (red) and SM22α (green) in the primary rat VSMCs cultured with or without ox-LDL (10 μg/ml) for 24 h. Nuclei were stained with DAPI (blue). n = 3; the scale bar represents 50 μm. M, N: Western blot analysis (M) and quantification (N) for the expression of CD68, α-SMA, and SM22α in the primary rat VSMCs transfected with or without si-Slug, followed by or not ox-LDL (10 μg/ml for 24 h) treatment. n = 4; ∗*P* < 0.05, ∗∗*P* < 0.01. DAPI, 4′,6-diamidino-2-phenylindole.

Slug is an invasive and metastatic cancer-related transcription factor that has been observed to promote VSMCs dedifferentiation in atherosclerotic plaques (17). In this study, we found the increased protein levels of Slug in the aortas of HFD-fed ApoE^−/−^ mice compared with standard diet-fed mice (Fig. 4A, B). And the upregulated Slug is mainly expressed in CD68-positive VSMCs, which are resided in the middle layer of the atherosclerotic fibrous cap from HFD-fed ApoE^−/−^ mice (Fig. 4C, D). Also, expression of Slug was quantified by Western blot analysis and localized by immunofluorescence staining in ligated carotid arteries from ApoE^−/−^ mice. The ligated carotid arteries had a higher level of Slug compared with sham-operated arteries (Fig. 4E, F). And VSMCs from the neointima of the ligated carotid artery showed high expression of Slug, and these are the same cells shown to dedifferentiate, losing contractile markers and gaining macrophage-like markers (Fig. 4G, H).

Then, we detected the expression of Slug in the macrophage-like VSMCs induced by ox-LDL. The results showed that the protein and mRNA levels of Slug increased in a dose-dependent manner (Fig. 4I–K). The similar trends in Slug and SM22α levels were observed by immunofluorescence staining assays (Fig. 4L). After we used the Si-Slug to knockdown the expression of Slug in VSMCs, the results showed that Slug knockdown reversed the phenotypic dedifferentiation of VSMCs induced by ox-LDL (Figs. 4M, N and supplemental Fig. S1F, G).

### RGMa promotes the dedifferentiation of ox-LDL-induced VSMCs by enhancing Slug

**Fig. 5:**
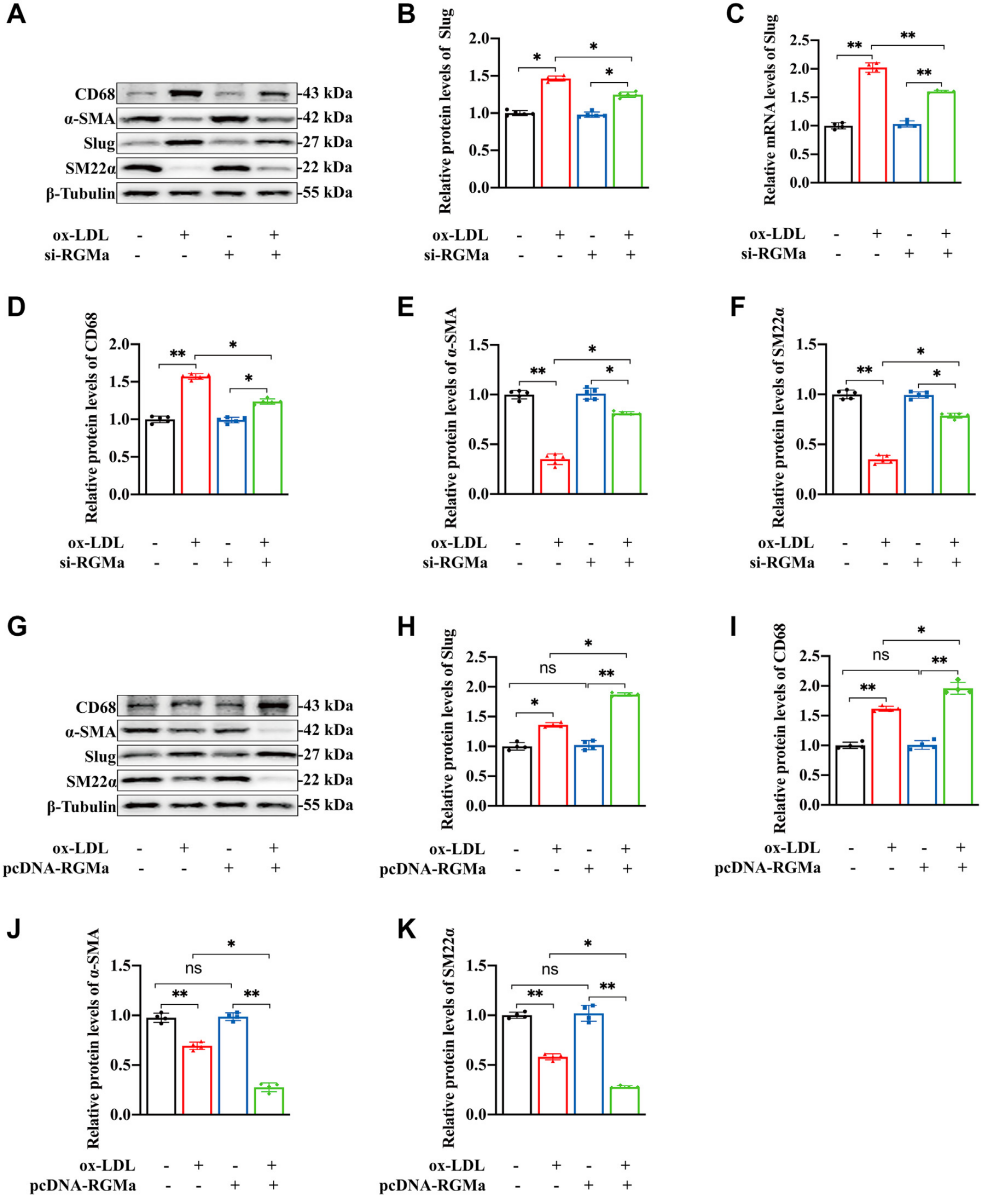

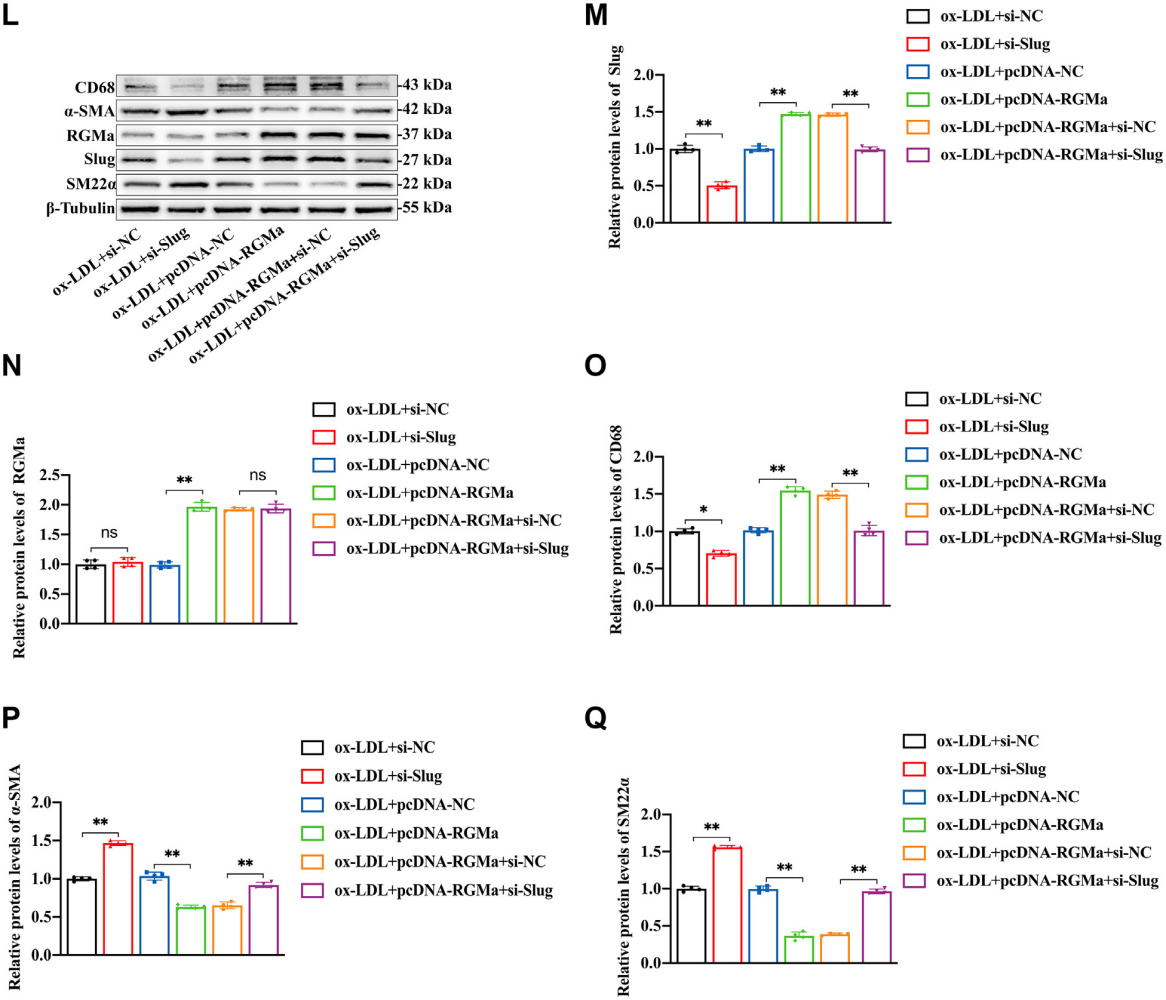
Slug knockdown reverses the dedifferentiation of ox-LDL-induced VSMCs promoted by RGMa overexpression. A–F: Western blot analysis (A) and quantification for the relative protein expression of Slug (B), CD68 (D), α-SMA (E), and SM22α (F), and the mRNA expression of Slug (C) in the primary rat VSMCs transfected with si-RGMa or si-NC, followed by or not 10 μg/ml ox-LDL for 24 h. n = 5; ∗*P* < 0.05, ∗∗*P* < 0.01. G–K: Western blot analysis (G) and quantification for the relative protein expression of Slug (H), CD68 (I), α-SMA (J), and SM22α (K) in the ox-LDL-induced primary rat VSMCs treated with pcDNA-RGMa or pcDNA-NC. n = 4; ∗*P* < 0.05, ∗∗*P* < 0.01, ns indicates not significant. L–Q: Western blot analysis (L) and quantification for the relative protein expression of Slug (M), RGMa (N), CD68 (O), α-SMA (P), and SM22α (Q) in the primary rat VSMCs treated with different conditions (ox-LDL + si-NC, ox-LDL + si-Slug, ox-LDL + pcDNA-NC, ox-LDL + pcDNA-RGMa, ox-LDL + pcDNA-RGMa + si-NC, and ox-LDL + pcDNA-RGMa + si-Slug). n = 4; ∗*P* < 0.05, ∗∗*P* < 0.01, ns indicates not significant.

The possibility of RGMa regulation of Slug was explored. Firstly, the protein and mRNA expression of Slug were found to decrease in the ox-LDL-induced VSMCs in which RGMa was silenced (Fig. 5A–C), and these changes were accompanied by increased protein levels of the VSMCs contractile markers and decreased protein levels of macrophage-like marker (Fig. 5D–F). In addition, overexpression of RGMa in the ox-LDL-treated VSMCs (supplemental Fig. S1H, I) upregulated the expression of Slug (Fig. 5G, H) and promoted the dedifferentiation of contractile VSMCs into a macrophage-like phenotype (Fig. 5I–K). Then the pcDNA-RGMa and si-Slug were transfected into ox-LDL-treated VSMCs to investigate whether Slug knockdown reversed the dedifferentiation of ox-LDL-induced VSMCs resulting from RGMa overexpression. Western blot analysis showed that overexpression of RGMa in ox-LDL-treated VSMCs promoted the dedifferentiation of contractile VSMCs, manifested by increased macrophage marker (CD68) and decreased VSMCs contractile markers (SM22α and α-SMA). But this effect was reversed by Slug knockdown and manifested by decreased macrophage marker and increased contractile markers (Fig. 5L–Q). Therefore, RGMa promotes the dedifferentiation of contractile VSMCs via enhancing the effect of Slug.

### RGMa enhances Slug in the dedifferentiation of VSMCs through the MEK1/2-ERK1/2 signaling pathway

**Fig. 6:**
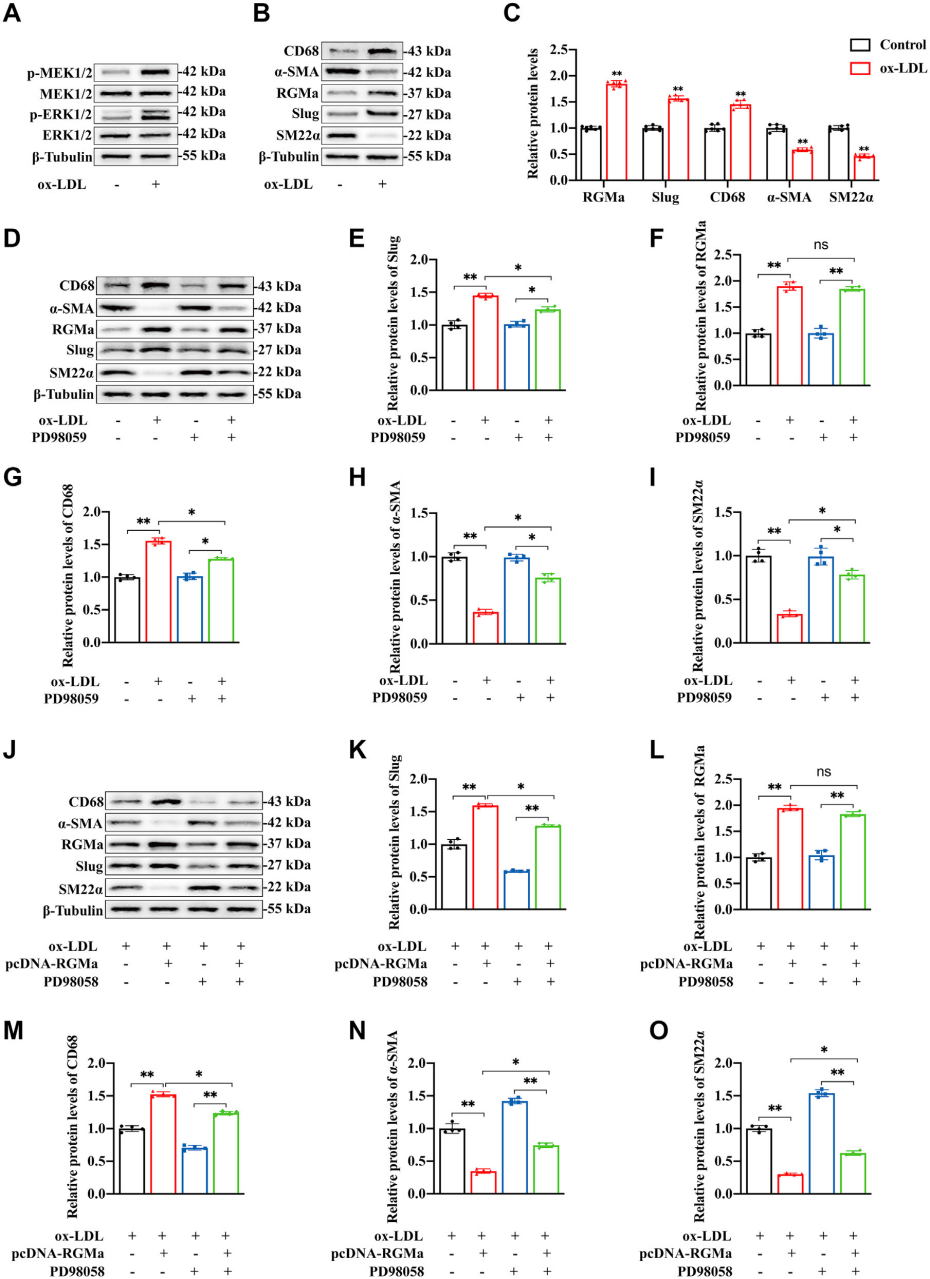
RGMa enhances Slug through MEK1/2-ERK1/2 signaling pathway. A: Western blot analysis for the expression of p-ERK1/2, ERK1/2, p-MEK1/2, and MEK1/2 in the primary rat VSMCs treated with 10 μg/ml ox-LDL for 24 h. n = 6. B, C: Western blot analysis (B) and quantification (C) for the expression of RGMa, Slug, CD68, α-SMA, and SM22α in the VSMCs treated with 10 μg/ml ox-LDL for 24 h. n = 6; ∗∗*P* < 0.01. D–I: Western blot analysis (D) and quantification for the expression of Slug (E), RGMa (F), CD68 (G), α-SMA (H), and SM22α (I) in the VSMCs treated with PD98059 for 2 h and/or followed by 10 μg/ml ox-LDL for 24 h. n = 4; ∗*P* < 0.05, ∗∗*P* < 0.01; ns indicates not significant. J–O: Western blot analysis (J) and quantification for the expression of Slug (K), RGMa (L), CD68 (M), α-SMA (N), and SM22α (O) in primary rat VSMCs treated with different conditions (ox-LDL + pcDNA-NC, ox-LDL + pcDNA-RGMa, ox-LDL + pcDNA-NC + PD98059, and ox-LDL + pcDNA-RGMa + PD98059). n = 4; ∗*P* < 0.05, ∗∗*P* < 0.01, ns indicates not significant.

Firstly, we found that the addition of ox-LDL promoted the dedifferentiation of contractile VSMCs into a macrophage-like phenotype, and these changes accompanied by the increase of RGMa and Slug, the phosphorylation of MEK1/2 and ERK1/2 (Fig. 6A–C). While the MEK-specific inhibitor, PD98059, impeded the expression of Slug in ox-LDL-treated VSMCs without change in RGMa (Fig. 6D–F). PD98059 also inhibited the dedifferentiation of ox-LDL-treated VSMCs, manifested by decreased CD68 and increased levels of α-SMA and SM22α (Fig. 6G–I). Then, to further explore whether the MEK1/2 and ERK1/2 pathway mediate the regulation of Slug by RGMa and thus participate in the dedifferentiation of contractile VSMCs, we used pcDNA-RGMa and PD98059 to overexpress RGMa and inhibit the activation of MEK1/2 and ERK1/2 pathway in ox-LDL-treated VSMCs. And Western blot analysis showed that RGMa overexpression increased the expression of Slug and promoted the dedifferentiation of ox-LDL-treated VSMCs, but this effect was reversed by MEK-specific inhibitor (Fig. 6J–O). These results indicate that MEK1/2-ERK1/2 signaling pathway mediates the effect of RGMa on Slug to promote dedifferentiation of contractile VSMCs into a macrophage-like phenotype.

## Discussion

It has been demonstrated that the dedifferentiation of contractile VSMCs into synthetic phenotypes, especially a macrophage-like phenotype, promotes the vulnerability of atherosclerotic plaques and aggravates restenosis caused by mechanical injury (31). However, the underlying molecular mechanisms of this complex pathophysiological process remain to be elucidated. The current study demonstrated the role of RGMa in regulating dedifferentiation of the contractile VSMCs into macrophage-like cells both in vivo and in vitro, thereby promoting the progression of atherosclerosis and intimal remodeling after injury.

Circulating BMP9 and BMP10 have been shown to directly interact with their receptors to maintain the contractile state of VSMCs in the pulmonary circulation (16). Signaling of RGMa-neogenin in infiltrating macrophages has been demonstrated to induce neutrophil-related inflammatory lesions (14). Thus, the possibility is exposed that RGMa, acts as both a co-receptor of BMP and a neogenin ligand, participates in the phenotypic transformation of VSMCs into macrophage-like cells. A high level of RGMa expression in the macrophage-like VSMCs of advanced atherosclerotic plaques and the neointima of ligated carotid arteries was observed during the current study. It is noteworthy that high RGMa expression occurs in the middle layer of fibrous cap where it is closely associated with the stability of advanced atherosclerotic plaques. Induction of VSMCs apoptosis, necrosis, and senescence in the plaque’s middle layer may reduce the protection of the fibrous cap and enlarge the necrotic lipid-rich center, resulting in the rupture of the unstable atherosclerotic plaques. Silencing of RGMa in VSMCs, both in vivo and in vitro, reverses the ox-LDL-induced dedifferentiation of contractile VSMCs and reduces neointimal thickness after injury. However, the proposed role of RGMa in promoting VSMCs dedifferentiation into macrophage-like cells seems to be at odds with our recent report of reduced plasma RGMa in stroke patients with atherosclerosis (18). We speculate it is possible that the reduced plasma RGMa results from a negative feedback loop, which reduces the formation of macrophage-like VSMCs in atherosclerosis. It may also be that the occurrence of the stroke, in itself, affects the RGMa level in plasma. Moreover, the current study focused on RGMa expression in VSMCs-derived macrophages within advanced atherosclerotic plaques, rather than blood-derived macrophages, which makes comparison difficult.

Slug has been shown to regulate EMT/EndMT through canonical SMAD pathways stimulated by TGF-β1 (23, 24). In addition, under the stimulation of ox-LDL and platelet-derived growth factor-BB, Slug promotes transdifferentiation of rat VSMCs into an inflammatory phenotype (25). Yan *et al.* (32) have identified RGMa as a suppressor of cancer metastasis and shown that knockdown of RGMa promoted EMT by suppressing E-cadherin and initiating Slug, vimentin, and β-catenin in breast cancer cells. The current study sought to ascertain whether RGMa promoted dedifferentiation of contractile VSMCs by enhancing the role of Slug. Consistent with the expression of RGMa, Slug is also observed in the macrophage-like VSMCs of the fibrous cap and the neointima of ligated carotid arteries. In vitro, Slug expression increased gradually in primary rat VSMCs induced by gradient ox-LDL and decreased in ox-LDL-treated VSMCs in which RGMa was silenced. Slug was also increased in RGMa-overexpressing VSMCs subjected to ox-LDL stimulation. Indeed, silencing Slug rescued the phenotypic dedifferentiation of ox-LDL-treated VSMCs caused by overexpression of RGMa. A previous study observed reduced RGMa enhanced the role of Slug in breast cancer cells that resulted in promotion of aggressive metastasis (32). Two possible explanations for the different changes of RGMa in breast cancer cells and VSMCs may be proposed. First, the origins of these two kinds of cells are quite different, the former originating from epithelial and the latter from mesenchymal cells. Second, cells in different states migrate in opposite directions, breast cancer cells in EMT migrate from the endothelial to the mesenchymal layer, whereas the dedifferentiated VSMCs migrate from vessel media to intima. Ledard *et al.* (25) reported that when ERK1/2 is activated in ox-LDL-induced rat VSMCs, Slug is stabilized and promotes transformation of VSMCs toward an inflammatory phenotype. RGMa also regulated the expression of chemotactic cytokines in macrophages accompanied by ERK1/2 phosphorylation (14). The current study found that ox-LDL-stimulated VSMCs upregulated the expression of Slug and was accompanied by MEK1/2 and ERK1/2 phosphorylation. Conversely, the MEK inhibitor, PD98059, downregulated Slug expression, thereby inhibiting markers of the macrophage-like state of VSMCs. Thus, during the dedifferentiation of VSMCs from their contractile to their macrophage-like phenotype, the RGMa regulation of Slug appears to be mediated by the MEK1/2-ERK1/2 signaling pathway.

The proliferation and migration of VSMCs are key steps in the progression of atherosclerosis and neointimal remodeling after injury. During the acute inflammatory responses, RGMa acts as an endogenous inhibitor of leukocyte chemotaxis to inhibit leukocyte migration and mitigate inflammation (33). However, during the chronic phase of inflammatory diseases, RGMa promotes T-cell proliferation and increases the secretion of interferon (34). The current study found that inhibition of RGMa reduced the migration and proliferation of ox-LDL-induced VSMCs. RGMa may sustain the chronic inflammatory response through promoting migration and proliferation in dedifferentiated VSMCs. Such findings are consistent with the observations concerning netrin-1 (35) and Sema3E (36) in atherosclerosis.

Reducing the area of atherosclerotic plaques does not necessarily prevent the occurrence of cardiovascular diseases but rather may lead to formation of unstable advanced plaques because of compensation for the absence of contractile VSMCs by other cell types. This observation illustrates that it is important to understand the whole process and underlying mechanisms from plaques formation to plaques rupture and repair in atherosclerosis, not only the role of inhibition of VSMCs phenotypic dedifferentiation.

The current study has some limitations. There is no generally acknowledged animal model of vulnerable atherosclerotic plaques, which limits observations to locational and quantitative analysis of type V atherosclerotic plaques in the aortas of ApoE^−/−^ mice without interventions.

In summary, the present study found that RGMa promoted dedifferentiation of VSMCs into macrophage-like cells by enhancing the role of Slug via the MEK-ERK1/2 signaling pathway. This result was to increase the vulnerability of atherosclerotic plaques and accelerate neointimal remodeling after arterial injury. Thus, inhibition of RGMa may reduce VSMCs dedifferentiation, exposing a therapeutic strategy for atherosclerotic plaques prone to rupture and restenosis following mechanical injury.

## Data availability

Data used in this study are available from Xiaofan Yuan by request (E-mail: 592604389@qq.com).

## Supplemental data

This article contains supplemental data.

## Conflict of interest

The authors declare that they have no conflicts of interest with the contents of this article.
